# The Alberta Ambassador Program: delivering Health Technology Assessment results to rural practitioners

**DOI:** 10.1186/1472-6920-6-21

**Published:** 2006-03-31

**Authors:** Saifudin Rashiq, Pamela Barton, Christa Harstall, Donald Schopflocher, Paul Taenzer

**Affiliations:** 1 Division of Pain Medicine, Department of Anesthesiology and Pain Medicine, University of Alberta, Edmonton AB, Canada; 2 Division of Physical Medicine and Rehabilitation, University of Calgary, Calgary AB, Canada; 3 Health Technology Assessment Unit, Alberta Heritage Foundation for Medical Research, Edmonton AB, Canada; 4 Health Surveillance, Alberta Health and Wellness, Edmonton AB, Canada; 5 Calgary Chronic Pain Centre, Calgary Health Region, Calgary AB, Canada

## Abstract

**Background:**

The purpose of Health Technology Assessment (HTA) is to make the best possible summary of the evidence regarding specific health interventions in order to influence health care and policy decisions. The need for decision makers to find relevant HTA data when it is needed is a barrier to its usefulness. These barriers are highest in rural areas and amongst isolated practitioners.

**Methods:**

A multidisciplinary team developed an interactive case-based instructional strategy on the topic of chronic non-cancer pain (CNCP) management using clinical evidence derived by HTA. The evidence for each of 18 CNCP interventions was distilled into single-sheet summaries. Clinicians and HTA specialists ('Ambassadors') conducted 11 two-hour interactive sessions on CNCP in eight of Alberta's nine health regions. Pre- and post-session evaluations were conducted.

**Results:**

The sessions were attended by 130 individuals representing 14 health and administrative disciplines. The ambassador model was well received. The use of content experts as ambassadors was highly rated. The educational strategy was judged to be effective. Awareness of the best evidence in CNCP management was increased. Although some participants reported practice changes as a result of the workshops, the program was not designed to measure changes in patient outcome.

**Conclusion:**

The ambassador program was successful in increasing awareness of the best evidence in CNCP management, and positively influenced treatment decisions. Its teaching methods were felt to be unique and innovative by participants. Its methods could be applied to other clinical content areas in order to increase the uptake of the results of HTA.

## Background

The need for practitioners to stay abreast of the medical literature is critical, but tools to actively support this goal are few. One response is Health Technology Assessment (HTA), a systematic search, assessment and summary of the extant literature on a specific health intervention or policy, analyzed both for outcome and the quality of the evidence itself. The goal of HTA is to produce credible information that policy makers and clinicians can use to guide clinical decision making and healthcare system change.

HTA reports or systematic reviews are usually generated in response to a specific question from an interested party but the information generated may be useful to other practitioners and organizations that may not be aware of its existence. An important goal of the HTA Unit of the Alberta Heritage Foundation for Medical Research (AHFMR) has therefore been to find better ways of disseminating HTA evidence.

The Alberta Ambassador Program was designed to test a specific HTA dissemination approach, based on an established Swedish strategy which seeks to promote changes in practice through education at the regional and local levels. Initiated in 1996, the Swedish Ambassador Program consists of a network of 40 permanent Ambassadors with at least one in each of Sweden's counties. These are clinicians who are also local opinion leaders. They disseminate HTA reports and promote research based decision making. Ambassadors receive centralized seminar-based training in HTA and are regularly updated on ongoing projects. In one region, 90 percent of physicians were aware of the existence of The Swedish Council on HTA, 40 percent were aware of the Ambassador Program, 75 percent felt that the program should continue and 50 percent stated they had made practical use of the information[1].

In our adaptation of the Swedish model, Ambassadors acted as both facilitators and content experts in interactive educational sessions for clinicians and decision makers.

The clinical topic selected for the project was chronic non-cancer pain (CNCP) management. The prevalence of CNCP ranges between 10% and 55% [2]. CNCP is burdensome in terms of personal suffering, impact on quality of life and loss of economic productivity [3]. Most health professionals have little training in treating CNCP. They tend to underutilize effective treatments and to over-use ineffective and hazardous modalities in CNCP, as in other areas of medicine [4].

Our primary objectives were therefore:

1. Develop and test a new model of HTA knowledge transfer

2. Increase awareness of the best evidence in CNCP management.

3. Change attitudes towards the management of CNCP

4. Steer practice towards the use of research evidence in the management of CNCP.

Secondary objectives were:

1. Promote awareness of AHFMR's HTA function;

2. Identify other areas in CNCP management where HTA evidence would be useful to Alberta health care providers.

3. Facilitate the development of a network of clinicians interested in CNCP

## Methods

The Calgary Health Region Conjoint Health Research Ethics Board approved the protocol.

The project's core concept was that experts in CNCP management and HTA would travel to each of the health regions in the province to translate the findings of relevant systematic reviews into meaningful messages for clinicians and policy makers. Chronic low back pain (CLBP) was selected as the subtype of CNCP on which the sessions were to be focused because it is an important clinical problem in primary care, and because it is the CNCP condition that has been researched most extensively.

We asked the Medical Director of each of Alberta's nine health regions to identify 10 local clinicians and/or policy makers who would be most likely to become local opinion leaders in CNCP management themselves, either because of a known interest in CNCP or an overlapping administrative mandate. We requested that half of the identified group be physicians but did not enforce this rigidly.

Our HTA specialists collated evidence on 18 possible treatments for CNCP. Thirteen of these were specific to CLBP (Table 1). These were selected by a panel of pain specialists and primary care physicians as being most likely to be applicable in primary care or a rural hospital. Tertiary-care-specific interventions were avoided. The evidence for each intervention was compiled into single-sheet summaries, known as '*Evidence-in-Brief*'. Each consists of a description of the best evidence available for the intervention, a categorical assessment of the quality and strength of the evidence, summaries of what is known and unknown about the intervention and finally, pragmatic recommendations from our group of clinical experts about the utility of the intervention. The summaries were externally reviewed. An example is shown (figure 1)

The teaching strategy was developed by a team consisting of clinicians, HTA specialists, medical educators, Clinical Practice Guidelines experts and communications experts. A list of characteristics that were most likely to increase the effectiveness of knowledge transfer was compiled (Table 2). Interactive workshops were designed that adhered to as many of these principles as possible. The final design was pilot-tested and refined.

Each workshop was conducted by a pain specialist ('Clinical Ambassador') and an HTA specialist ('Research Ambassador'). The workshop followed a set order:

(i) Completion of a pre-workshop evaluation.

(ii) An opportunity for participants to identify their own particular challenges in managing CNCP.

(iii) A presentation from the Research Ambassador about the role of the HTA Unit and how the evidence was collected.

(iv) The case study discussion, which took up the majority of the two-hour workshop. The Clinical Ambassador presented a standardized hypothetical case study of CLBP. Participants were then encouraged to propose different treatments. As each suggested treatment was brought forward, the Ambassadors produced copies of the appropriate '*Evidence in Brief*, summary sheet for everyone. The Clinical Ambassador then moderated a discussion of the benefits and drawbacks of the proposed treatment, its place in the sequence of treatments that might properly be applied to the case, and the availability of such treatment in the area. The participant group determined the sequence of the treatment algorithm. All the '*Evidence-in-Brief*' summaries were distributed at the end of the session, even if the particular intervention was not suggested during the case study. Participants were explicitly encouraged to copy the sheets for redistribution to others and a website was established to allow participants and others to download them. The website was to be updated every four months over a one-year period.

(v) The opportunity to develop an action plan for improving the situation within local resource and logistic constraints. The information from the action plans was collated and sent back to participants in written form within two weeks of the workshop.

An independent evaluator solicited participants' and Ambassadors' opinions on various aspects of the process by means of pre-(immediately prior to the workshop) and post-(six weeks afterwards) workshop questionnaires and interviews. Simple descriptive statistics were calculated for quantitative responses, while qualitative data (from open-ended items) were analyzed using traditional content analysis techniques [5].

## Results

130 participants attended 11 workshops between October and December 2004. At least one workshop took place in 8 of Alberta's 9 health regions. Attendance per region averaged 16 participants (range 8 to 28).

27% of participants were nurses, 21% physicians, 18% physical or occupational therapists, 17% administrators, 9% pharmacists, and 7% were psychologists, mental health or social workers. They reported a median of 20 encounters with chronic pain patients during a typical three-month period (range 1 to 1000). They reported a high degree of interest in CNCP.

All participants completed the pre-workshop survey. 79 participants (60.8%) completed the post-workshop survey.

In the pre-workshop survey, 77% believed that they had at least some ability to influence their colleagues, while 60% reported having at least some ability to influence administrators. 78% attended in order to gather information on CNCP management while 75% attended to gather information on best evidence (more than one response was possible), 29% attended because of the opportunity to network, and 26% attended because of the expertise of the Ambassadors.

67% stated that there was either some or a great deal of encouragement from their organizational leadership to use new knowledge. However, fewer described the existence of a culture of knowledge sharing (51%) or the necessary infrastructure for that purpose (32%) in their own organizations.

In the post-workshop survey, 78 (99%) indicated that the workshops had been a useful way of linking research to practice. In most areas relating to content and presentation, satisfaction scores were high (Table 3). The '*Evidence-in-Brief*' summaries were particularly well received. The action planning component was the least satisfactory: 30% of respondents reported that no action plan had been developed, mostly because of insufficient time in the session. 39 respondents (50%) reported making plans to improve CNCP management in some form where none had existed previously.

We determined the impact of the workshops on participants' perceptions of their knowledge about CNCP by asking them how much knowledge they had in 5 sample topic areas (Table 4). Perceived knowledge rose after the workshop, but. we did not test this directly.

The goal of onward dissemination of the material distributed in the sessions was well met. 62 (80%) of follow-up survey respondents had done so, to nurses (21%), physicians (16%) and physical therapists (14%), other health professionals, administrators, patients and pharmaceutical representatives. 85% of respondents indicated that they had or planned to access the website. The most popular download was a document that outlined the evidence gathering process, followed by the sheets on muscle relaxants, exercise therapy and long-acting opioids. Every content topic was accessed to some degree. Downloads peaked while the workshops were in progress. One year after completion of the last workshop, the site was being accessed an average of 19 times a day.

67 respondents (87%) indicated their intent to follow up with AHFMR's HTA Unit in some way, indicating that the goal of raising awareness of the organization's existence and function was met. 31% of respondents planned to initiate requests for HTA assessments of their own. The areas most frequently identified as being amenable to this kind of educational strategy were chronic headaches, arthritis, fibromyalgia, medications, alternative therapies and diabetes, but several respondents indicated that it would work well in any condition. 35% reported that the workshop had resulted in important changes in the way they manage CNCP.

## Discussion

We delivered high-quality, credible research evidence about CNCP management to clinicians and policy-makers throughout Alberta. We exceeded our target number of participants and provided an educational experience that exceeded their expectations.

In trying to select the most effective educational approach we made certain choices. Arguably the most important, and certainly the most difficult to execute, was that between the desire of the HTA specialists in our group to restrict our offerings to only those of the highest methodological quality, and the clinicians' wish to present as broad a range of therapeutic options for discussion as possible. This dichotomy is particularly strong in CNCP, where the quality of much of the evidence base is low.

The highly abbreviated summaries that appear on the '*Evidence in Brief*' sheets were well received. The acceptability of this degree of abbreviation may have been enhanced by the perceived credibility of the ambassadors as content and methodology experts. The credibility of the source has been shown to play a major role in the way physicians use information to make treatment decisions [6].

The 61% response to our follow-up questionnaire exceeds the 50% generally considered acceptable in this type of investigation [7] but we cannot rule out the possibility that the non-respondent group had a different assessment of the workshop.

Our knowledge transfer product was a hybrid but contained several elements that were identified in a recent review of randomized trials as being effective elements of continuing medical education, namely two-way communication (between educator and audience), small group format, personal delivery of printed materials and the use of locally respected clinicians as educators [8]. Our decision to use case-based context arose from its demonstrated superiority over text-based content in increasing participants' knowledge [9].

We offered information on 18 CNCP treatments, most of which focused on chronic low back pain. The ability to deliver wider selection of content might have made the sessions even more appealing, but would have made even greater demands of our HTA specialists and clinical ambassadors, in the context of an already highly resource-intensive program.

We did not change interest in or attitudes toward the importance of CNCP as a health issue. We think that this merely means that those who already regarded CNCP as an important health care issue were preferentially selected for our sessions.

Our measure of participants' knowledge of CNCP was a self-reported surrogate measure rather than a knowledge test *per se*. This is a weak measure and therefore only suggests rather than verifies that increases in knowledge occurred.

Known obstacles to the effectiveness of research transfer in health services include ineffective continuing education programs, poor access to best evidence guidelines and organizational barriers [10]. Our strategy addressed the first two factors, but we could not control factors at play in participants' own institutions, such as a lack of institutional had a culture of or infrastructure for fostering new knowledge. In addition, we were compelled to conduct our program in a relatively short time frame. Buckley *et al *[11] noted that even when the intentions of workshop participants have changed from pre- to post-test, even a twelve-week interval (twice as long as ours) may be insufficient to observe a subsequent change in practice.

Several participants indicated that they were surprised by the non-conventional teaching methods we employed. It is known that when participants know what to expect in the training session, they learn more and are better able to implement what they learned in practice [12]. Knowing what to expect might have improved participants' learning still further.

We would have preferred to see a greater number of physicians in the participant group, in the belief that physicians are better placed to influence the course of care than other professions. Despite our best efforts, we may not have offered sufficiently compelling reasons for the prototypical overworked rural physician to attend. In this respect, our program differed significantly from the Swedish model, which is targeted exclusively at physicians.

Of the session components, the action planning was the least successful. We had hoped that the acquisition of new content knowledge on CNCP would rapidly result in the synthesis of ideas and plans for its clinical implementation. In reality, learning and strategic planning are different activities that, while related, require different participants, skills and logistics to be successful.

The Alberta Ambassador Program in CNCP therefore succeeded in achieving five of its seven objectives: a new research transfer model was developed and tested, the best evidence in CNCP management was promulgated widely in the province, awareness of the existence of AHFMR's HTA unit was increased, other possible areas for HTA and knowledge transfer activities were identified and some changes in participants' practice in the area of CNCP were reported. We were unable to document significant changes in clinician attitude towards CNCP or promote the development of action plans or interest networks. We suggest that this is a model of health care education that has significant potential to advance the usefulness of HTA in general.

## Conclusion

1. The results of Health Technology Assessment can change clinical practice and positively affect health policy decisions.

2. Highly abbreviated summaries of HTA evidence are useful to clinicians.

3. Interactive sessions delivered locally are a productive way of transferring HTA knowledge to practitioners in rural regions.

## Authors' contributions

PT first articulated the study idea. All authors participated in the design and execution of the study in equal measure. SR wrote the manuscript which was critically revised by all others.

## Pre-publication history

The pre-publication history for this paper can be accessed here:



## Figures and Tables

**Table 1:** 'Evidence in Brief' summaries prepared for the workshops.

*Focused on CLBP in particular*	*Focused on CNCP in general*
Acupuncture Antidepressants Behavioral Therapy COX-2 Inhibitors (etoricoxib) Epidural Steroid Injections Exercise Therapy Massage Therapy Muscle Relaxants Multidisciplinary Pain Programs Non-steroidal Anti-inflammatory Drugs (NSAIDs) Prolotherapy Injections Spinal Manipulative Therapy Transcutaneous Electrical Nerve Stimulation(TENS)	Cannabis or Cannabinoids COX-2 inhibitors for osteoarthritis and rheumatoid arthritis Gabapentin Long-acting Opioids Trigger Point Injections

**Table 2:** Factors incorporated into workshop design in order to maximize knowledge transfer.

▪ Conduct workshops close to the participants' place of work
▪ Challenge participants to actively engage the material and problem solve by using simulations, stories, or case studies.
▪ Allow participants to affect the direction of the workshop.
▪ Keep the workshops as short as possible
▪ Keep group size small
▪ Encourage a multi-disciplinary mix of participants
▪ Minimize the didactic component of the workshop
▪ Make all printed materials as concise as possible.
▪ Include a powerful, positive closing message.
▪ Provide immediate written feedback.
▪ Offer continuing education credits for participation.
▪ Offer refreshments.
▪ Connect with participants afterwards to reinforce learning.

**Table 3:** Participants' evaluation of Workshop Structure and content

**Item**	**n**	**Median Rating**	**N (%)**				
			
			**Not at all satisfied 1**	**2**	**3**	**4**	**Very satisfied 5**
Presentation by Ambassador	81	4	0	0	11 (14%)	37 (46%)	33 (41%)
Interactive approach	80	4	0	3 (4%)	11 (14%)	33 (41%)	33 (41%)
Case study	77	4	1 (1%)	3 (4%)	12 (16%)	32 (42%)	29 (38%)
One-page summaries	81	5	0	1 (1%)	5 (6%)	25 (31%)	50 (62%)
Action planning	76	4	1 (1%)	6 (8%)	22 (29%)	33 (43%)	14 (18%)

			**Strongly disagree 1**	**2**	**3**	**4**	**Strongly agree 5**

The Ambassadors are a source of knowledge I respect	79	4.5	0	0	6 (8%)	34 (43%)	39 (50%)
Workshop content was relevant for my organization/environment	73	4	0	3 (4%)	8 (10%)	34 (43%)	35 (44%)
Material was presented in language that held meaning for me	81	4	0	0	7 (9%)	41 (51%)	33 (41%)
Content was relevant for my practice	79	4	0	5 (6%)	10 (13%)	34 (43%)	30 (38%)
Workshop met my information needs	81	4	3 (4%)	3 (4%)	18 (22%)	35 (43%)	22 (27%)

**Table 4:** Participant Change on Chronic Pain Knowledge Questions. (1 = *little or no knowledge *and 5 = *a great deal of knowledge*)

**Item**	**N**	**Mean Rating at Pre-test**	**Mean Rating at Post-test**
Whether physical therapy for chronic pain is better than exercise	71	2.9	3.9*
When I should refer to a multidisciplinary pain center	65	3.1	3.9*
How psychotherapy can help patient chronic low back pain.	74	2.7	3.6*
The evidence for cannabinoid use in chronic pain	74	2.3	3.4*
When I should send my chronic back patients for spinal manipulation.	64	2.4	3.4*

* p < .001
